# Prevalence and risk factors for High-Risk Human Papillomavirus (hrHPV) infection among HIV-infected and Uninfected Rwandan women: implications for hrHPV-based screening in Rwanda

**DOI:** 10.1186/1750-9378-9-40

**Published:** 2014-12-08

**Authors:** Jean d’Amour Sinayobye, Marc Sklar, Donald R Hoover, Qiuhu Shi, Jean Claude Dusingize, Mardge Cohen, Eugene Mutimura, Brenda Asiimwe-Kateera, Philip E Castle, Howard Strickler, Kathryn Anastos

**Affiliations:** Regional Alliance for Sustainable Development (RASD), P. O. Box 1544, Kigali, Rwanda; Albert Einstein College of Medicine, Bronx, NY USA; Rutgers University, New Brunswick, NJ USA; NY Medical College, Valhalla, NY USA; John Stroger (Cook County) Hospital, Chicago, IL USA; Global Coalition Against Cervical Cancer, Arlington, VA USA; University of Rwanda, College of Medicine and Health Sciences, Butare, Rwanda

**Keywords:** HPV, HIV, Cervical cancer, Screening

## Abstract

**Background:**

New World Health Organization guidelines recommend high-risk human papillomavirus (hrHPV) screen-and-treat strategies for cervical cancer prevention. We describe risk of, and risk factors for, testing hrHPV positive in a pilot study of hrHPV screen-and-treat conducted in Rwanda.

**Methods:**

A total of 2,964 women, 1,289 HIV-infected (HIV [+]) and 1,675 HIV-uninfected (HIV [-]), aged 30-60 years and living in Rwanda were enrolled in 2010. Cervical specimens were collected and tested by careHPV, a DNA test for a pool of 14 hrHPV types. Prevalence with binomial 95% confidence intervals (95% CI) and determinants of testing hrHPV positive were calculated.

**Results:**

hrHPV prevalence was higher in HIV [+] (31.8%, 95% CI = 29.2-34.4%) than HIV [-] women (8.2%, 95% CI = 6.7-9.8%; P < 0.0001). Among HIV [+] women, there was a significant trend (p_trend_ <0.001) of higher hrHPV prevalence with lower CD4 cell count, with the highest hrHPV prevalence among those with <200 CD4 cell counts (45.5%, 95% CI = 34.8-56.4%). In multivariate analysis of HIV [+] women, testing hrHPV positive was positively associated CD4 count of <200 cells/μL, history of 3 or more sexual partners, and history of using hormonal contraception, and negatively associated with older age. In HIV [-] women, testing hrHPV positive was negatively associated only with older age groups of 45-49 and 50-60 years and surprisingly was not associated with lifetime number of sexual partners.

**Conclusion:**

hrHPV prevalence is high in HIV [+], especially in women with the lowest CD4 cell counts, which may have implications for utilizing hrHPV-based screening strategies such as screen-and-treat in these high-risk subgroups.

## Introduction

Globally, cervical cancer is the third most common female cancer and cause of female cancer-related deaths, with an estimated 530,000 cases and 265,653 deaths annually, respectively [1]. In low- and middle-income countries (LMICs), cervical cancer is often the most common cancer, the leading cause of cancer-related mortality, and a leading cause of all-cause mortality in women due to a lack of preventive services. Cervical cancer constitutes 13% of all female cancers in LMICs [2, 3]. More than 85% of cervical cancer deaths occur in LMICs [3]. Approximately one quarter of cervical cancers and related mortality globally occur in Africa, and >90% of those occur in Sub-Saharan Africa, which has an overall age-standardized rate of cervical cancer incidence of 34.8 per 100,000.

Introduction of large-scale cervical cancer cytology-based screening programs, utilizing the Papanicolaou (Pap) smear has dramatically reduced cervical cancer incidence and mortality in developed countries [4]. For example, an estimated 45% of all cervical cancer was prevented over a 50-year period (1961-2010) by the introduction of cervical cytology-based screening in Denmark, Finland, Norway, and Sweden [5]. The cost and complexity of cytological screening and inadequate health infrastructure, in addition to the lack of human resource capacity, make this screening approach impractical and unsustainable in the developing world [6, 7].

Persistent cervical infection by approximately 12-15 carcinogenic or high-risk human papillomavirus (hrHPV) genotypes causes virtually all cervical cancer and its immediate precursors [8–10]. HPV genotype 16 (HPV16) causes approximately 55% and HPV18 causes approximately 15% of cervical cancers [11]. HrHPV infections are typically transient and clear or become undetectable within 1-2 years [8, 12]. Those hrHPV infections that persist for even one year [13] or two years [14] strongly predict the development of cervical precancer and cancer.

The discovery that persistent hrHPV is the cause of cervical cancer has led to technological developments, including molecular hrHPV testing for cervical cancer screening. Molecular hrHPV testing is more sensitive [15–20] and reliable [21–23] for detection of cervical intraepithelial neoplasia (CIN3), adenocarcinoma in situ (AIS), or invasive cervical cancer (≥CIN3) than Pap testing. The increased sensitivity of hrHPV testing over Pap testing for ≥ CIN3 translates into two important benefits: 1) earlier detection of all high grade lesions that if treated results in a reduced incidence of cervical cancer within 4-5 years [24] and related death within 8 years [25] and 2) greater reassurance against cancer (lower cancer risk) for many years following a negative result [24–28], which permits screening at an extended interval of 5-10 years. The World Health Organization (WHO) has recently recommended hrHPV testing and visual inspection after acetic acid (VIA) as alternatives to Pap testing for those countries that do not have high-coverage Pap testing [29]. If the resources are available, hrHPV testing is recommended, either alone or with VIA evaluation of hrHPV positives, although using VIA as a triage of hrHPV positives may significantly reduce the sensitivity of screening [29, 30].

Current U.S. Food and Drug Administration (FDA) approved hrHPV tests are too costly and complex to use in many settings in LMICs. To address the need for a simpler, lower-cost hrHPV test for LMICs, careHPV™ (Qiagen, Gaithersburg, MD, USA) was developed based on the same chemistry as its U.S. FDA approved predecessor, Hybrid Capture 2 (HC2; Qiagen). careHPV is a DNA test for a pool of 13 hrHPV genotypes (HPV16, 18, 31, 33, 35, 39, 45, 51, 52, 56, 58, 59, 68) and one possibly hrHPV genotype (HPV66). Previous studies have shown that careHPV has good sensitivity and specificity for cervical precancer and cancer that approaches HC2 [31, 32] and performs reasonably well using self-collected specimens [31–33].

Data from developing countries on the overall hrHPV infection prevalence and associated risk factors is imperative to develop and guide screening strategies particularly in Sub-Saharan Africa, which bears the greatest dual burden of human immunodeficiency virus (HIV) infection and cervical cancer. To date, information on hrHPV prevalence and associated risk factors in the HIV-infected population comes from a few African countries such as Kenya, Nigeria, Cameroun and South Africa [34–38]. There is uncertainty about how generalizable these data are to HPV infection patterns in other African settings with varying cultural and social dynamic characteristics.

HIV is known to be associated with higher prevalence and persistence of HPV infection [39, 40]. A previous report on 188 HIV-uninfected (HIV [-]) and 628 HIV-infected (HIV [+]) Rwandan women found ~5-fold higher prevalence of hrHPV in antiretroviral (ART)-naïve, HIV [+] compared to HIV [-] women. However, in this study, only women living in an urban setting were included, none of the HIV [+] women was receiving ART, and only a small number of HIV [-] women were recruited. Thus, the generalizability of those findings to Rwanda and other countries is limited.

In 2010, we conducted a sentinel evaluation of screen-and-treat strategies, prior to the launch of national program of hrHPV screen-and-treat in Rwanda [41] anticipated for 2014 and the endorsement of such a strategy by the WHO in late 2013 [29]. We recruited approximately 1,300 HIV [+] and 1,700 HIV [-] women into the evaluation. All women were screened by careHPV and VIA and was treated by cryotherapy if hrHPV or VIA positive (n.b., VIA was used as a screen and not as a triage for hrHPV-positive women.). At enrollment, we collected data on risk factors through an administered questionnaire. Here we present the prevalence of and risk factors for hrHPV by HIV status in these Rwandan women, which has implications for implementation of the careHPV-based screening program there.

## Methods

### Study population

**Figure 1:**
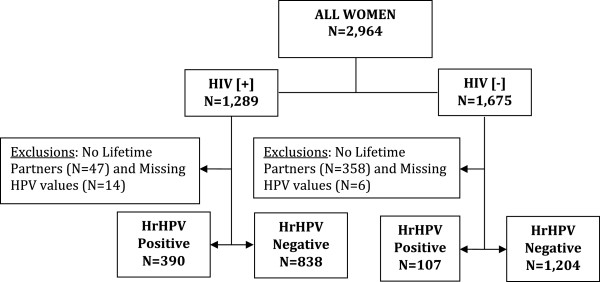
**Consort diagram for the 2,971 human immunodeficiency virus-infected (HIV [+]) and uninfected (HIV [-]) women aged 30-60 years and living in Rwanda enrolled and tested for high-risk human papillomavirus DNA by careHPV.**

This is a cross-sectional analysis of 2,964 women, 1,289 HIV [+] and 1,675 HIV [-], women aged 30-60 years, and living in Rwanda. Figure 1 presents a consort diagram of enrolled women and exclusions by HIV status. Women were recruited from Nduba and Jabana sectors served by the Nyacyonga Public Health Center under the Kibagabaga District Hospital in Gasabo district, with 1,000 of the HIV [+] women recruited from an HIV-dedicated clinic in Kigali. The study population was a mix of women living in urban and rural settings to increase the representativeness of the Rwandan population. Most of the recruitment was done through community-based outreach teams of Community Health workers (CHWs) who were trained as cervical cancer educators. Inclusion criteria were age 30-60 years, ability and willingness to give written informed consent for study procedures, have an HIV test, and blood drawn for CD4 cell count determination if found to be HIV positive. Past history of cervical cancer screening and/or being pregnant was an exclusion criterion.

The study protocol and written informed consent process were approved by the Rwanda National Ethics Committee and the Institutional Review Board of Montefiore Medical Center (Bronx, NY). Trained research teams gathered medical history focusing on reproductive and sexual health through a short interview from consenting women. The consent interview was done in Kinyarwanda (national Rwandan language), which was translated from the original English version by experts in the two languages and there were no specific exclusionary factors regarding language. A speculum exam was performed with the collection of an endocervical specimen using a “Christmas tree brush”, placed into **DCM™** medium (Qiagen) for careHPV testing.

All women whose HIV tests were positive had blood drawn for CD4 cell count, and were referred to a nearby public health center for CD4+ count determination and HIV care and treatment. HIV infection was diagnosed by a testing algorithm, which included 2 commercial HIV-1 antibodies enzyme-linked immunosorbent assay kits (HIV Vironostika, Netherlands, and Murex HIV-1.2, Oxford, UK). CD4 counts were determined with a flow-activated cell counter (Becton and Dickinson, Immunocytometry Systems, San Jose, CA, USA). The HIV infection and CD4 results were provided to the treatment site by the program outreach staff.

### Case definition and secondary variables

HrHPV infection, a dichotomous outcome variable, was defined as having a positive careHPV DNA test. We considered as potential covariates the following variables found to be related to testing hrHPV positive in previous studies or otherwise biologically plausible; HIV related immunological status (CD4), current age, menopausal status, age at sexual debut, age at first pregnancy, parity, history of malaria, tobacco use, hormonal contraceptive use, lifetime number of sexual partners, polygamous relationship, rape history, and ART use.

### Statistical analysis

Data were analyzed using STATA 11.1 (StataCorp LP, College Station, TX, 2010). Descriptive analyses including means, standard deviations, medians and interquartile ranges were performed for continuous variables, and percentages for categorical variables. This was stratified by HIV status and by CD4 count (≥500, >350-499, 200-349 and <200 cells/μl) for HIV-infected women. The same descriptive statistical analysis of baseline demographic and clinical characteristics were stratified both by HIV infected vs. uninfected women and by hrHPV status. Analysis of variance and non-parametric Kruskal Wallis tested for statistical differences of continuous variables across different HIV, CD4 and hrHPV infection groups. Chi-square tests and logistic regression analysis assessed the association of categorical variables (including HIV/CD4 status) with hrHPV infection categories. All covariates in Table 1 were considered for multivariable logistic regression models as they had been found to be associated with HPV infection in previous studies or because of biological plausibility. However, body mass index (BMI) and age at sexual initiation had a considerable amount of missing values and were thus excluded from multivariate analyses to prevent data loss.

**Table 1:** **Baseline Characteristics by HIV and Immunological level (CD4 count) in both HIV-infected (HIV [+]) and -uninfected {HIV [-]) women**

Parameters	HIV[-] N = 1,311	HIV[+] N = 1,228	p*	HIV[+] N = 1,228				p **
				CD4: ≥500	CD4:350-499	CD4:200-349	CD4:<200	
				N = 513	N = 346	N = 271	N = 88	
**Age, years, mean ± SD**	42.0 ± 8.2	40.0 ± 6.6	<0.001	39.8 ± 6.6	40.1 ± 6.7	40.8 ± 6.5	39.7 ± 6.5	0.21
**Age category, years, n (%)**			<0.001					0.17
**Age 30 – 34**	299 (22.8)	278 (22.6)		127 (24.8)	81 (23.4)	51 (18.8)	16 (18.2)	
**Age 35 – 39**	279 (21.3)	359 (29.3)		144 (28.1)	101 (29.2)	76 (28.0)	35 (39.8)	
**Age 40 – 44**	237 (18.1)	295 (24.0)		134 (26.1)	80 (23.1)	63 (23.3)	17 (19.3)	
**Age 45 – 49**	211 (16.1)	171 (13.9)		58 (11.3)	49 (14.2)	49 (18.1)	13 (14.8)	
**Age at sexual initiation, years, n (%)**			<0.001					0.01
**Age <14**	16 (4.2)	30 (10.2)		11 (10.0)	5 (5.1)	7 (11.9)	7 (28.0)	
**Age 14 – 25**	338 (89.7)	258 (87.8)		97 (89.0)	92 (93.9)	49 (83.0)	17 (68.0)	
**Age at first pregnancy, years ± SD**	21.1 ± 3.7	20.1 ± 3.6	<0.001	20.1 ± 3.6	20.3 ± 3.6	20.0 ± 3.7	19.4 ± 3.4	0.25
**Lifetime sexual partners**			<0.001					0.59
**1- 2 sexual partners**	1132 (86.4)	616 (50.2)		260 (50.7)	179 (51.7)	126 (46.5)	43 (48.9)	
**≥3 sexual partners**	179 (13.6)	612 (49.8)		253 (49.3)	167 (48.3)	145 (535)	45 (51.1)	
**Number of children delivered, n (%)**			<0.001					0.01
**0 – 2**	300 (23.7)	448 (39.6)		163 (33.9)	130 (41.3)	117 (47.8)	37 (44.6)	
**3 – 4**	500 (39.5)	464 (41.0)		211 (43.9)	122 (38.7)	93 (37.9)	33 (39.7)	
**5 – 12**	466 (36.8)	219 (19.4)		107 (22.2)	63 (20.0)	35 (14.3)	13 (15.7)	
**Rape, n (%)**			<0.001					0.46
**Yes**	187 (14.3)	409 (33.3)		164 (32.0)	124 (35.8)	85 (31.4)	33 (37.5)	
**No**	1123 (85.7)	819 (66.7)		349 (68.0)	222 (64.2)	186 (68.6)	55 (62.5)	
**Menopause, n (%)**			<0.001					0.58
**Yes**	219 (16.7)	111 (9.0)		50 (9.7)	26 (7.5)	28 (10.3)	7 (8.0)	
**No**	1092 (83.3)	1117 (91.0)		463 (90.3)	320 (92.5)	243 (89.7)	81 (92.0)	
**Polygamous relationship, n (%)**			0.01					0.81
**Yes**	60 (9.2)	34 (14.9)		15 (15.9)	8 (12.1)	8 (15.4)	3 (21.4)	
**No**	593 (90.8)	194 (85.1)		79 (84.1)	58 (87.9)	44 (84.6)	11 (78.6)	
**Ever used hormonal contraceptives, n (%)**			0.002					< 0.001
**Yes**	393 (30.1)	305 (24.8)		154 (30.1)	86 (24.9)	45 (16.6)	20 (22.7)	
**No**	912 (69.9)	922 (75.2)		358 (69.9)	260 (75.1)	226 (83.4)	68 (77.3)	
**Ever used tobacco, n (%)**			<0.001					0.57
**Yes**	221 (16.9)	57 (4.6)		25 (4.9)	11 (3.2)	13 (4.8)	5 (5.8)	
**No**	1089 (83.1)	1169 (95.4)		488 (95.1)	335 (96.8)	258 (95.2)	81 (94.2)	
**Antiretroviral therapy, n (%)**								< 0.001
**Yes**	N/A	948 (78.1)		342 (67.6)	273 (79.6)	274 (92.2)	82 (93.2)	
**No**	N/A	266 (21.9)		164 (32.4)	70 (20.4)	21 (7.8)	6 (6.8)	
**Malaria within 6 months, n (%)**			<0.001					0.28
**Yes**	185 (14.0)	95 (7.8)		47 (9.2)	23 (6.6)	16 (5.9)	5 (5.7)	
**No**	112 4(86.0)	1133 (92.2)		466 (90.8)	323 (93.4)	255 (94.1)	83 (94.3)	
**BMI, mean ± SD, kg/m** ^**^2**^	21.9 ± 3.7	22.6 ± 3.8	<0.001	23.0 ± 3.8	22.7 ± 3.8	22.1 ± 3.8	22.0 ± 3.4	0.01
**BMI, n (%), kg/m** ^**2**^			<0.001					0.38
**12.5 – 18.5**	136 (15.7)	135 (11.6)		44 (9.3)	41 (12.4)	38 (14.3)	11 (12.6)	
**18.5 – 21.0**	243 (28.1)	282 (24.3)		113 (23.8)	77 (23.3)	68 (25.6)	24 (27.6)	
**> 21 – 39.5**	486 (56.2)	744 (64.1)		318 (66.9)	212 (64.3)	160 (60.1)	52 (59.8)	
HrHPV infection, n (%)*			<0.001					0.02
**Yes**	107 (8.2)	390 (31.8)		156 (30.4)	103 (29.8)	91 (33.6)	40 (45.5)	
**No**	1,204 (91.8)	838 (68.2)		357 (69.6)	243 (70.3)	180 (66.4)	48 (54.5)	

SD, standard deviation and BMI, Body Mass Index. P*-value is for comparing HIV-uninfected to infected overall. P**-value is for comparing CD4 categories in HIV -infected women to different subjects’ parameters. For obtaining p-values chi-Square tests were used for categorical variables and ANOVA for continuous variables except: number of children born, where non-parametric Kruskal Wallis tests was used.

To avoid potential investigator bias by selecting or weighting variables for inclusion, stepwise selection logistic regression with p value to enter and stay of 0.10 was used to build two separate multivariate predictive models of hrHPV infection, one for HIV [+] and one for HIV [-] women. Odds Ratios (OR) and 95% confidence intervals (95% CI) were calculated as indicators of the magnitude of association and statistical significance of hrHPV infection.

## Results

Participants’ baseline demographic and clinical characteristics by HIV status and among HIV [+] immunological status (CD4+ cell count categories) are summarized (Table 1). Comparing HIV [+] and HIV [-] women, HIV [-] were older, older at sexual initiation, older at first pregnancy, had delivered more children, more likely to ever use hormonal contraceptives, more likely to ever use tobacco, have a prior episode of malaria, and had a lower BMI compared to HIV [+] women (p < 0.05 for all). By comparison, HIV [+] had more sexual partners in their lifetime, were more likely to have a polygamous relationship, reported a history of rape, and tested positive for hrHPV than HIV [-] women (p < 0.05 for all). Because the populations were distinct, we elected to conduct our analyses separately in HIV [+] and HIV [-] populations rather than combine populations.

Among HIV [+] women, women with lower CD4 counts (more immunosuppressed) had fewer children, were less likely to use hormonal contraceptives, had a lower BMI, and were more likely to test hrHPV positive.

Tables 2 and 3 summarize baseline demographic and clinical characteristics by hrHPV status in HIV [+] and HIV [-] women, respectively. Among HIV [+] women, hrHPV-infected women were younger, younger age at first pregnancy, had lower CD4 cell count, fewer lifetime sexual partners and less number of hormonal contraceptive (p <0.05 for all). Among HIV [-] women, hrHPV infected women were younger, and fewer post-menopausal status (p <0.05 for all).

**Table 2:** **Baseline characteristics by high-risk human papillomavirus (hrHPV) status, restricted to HIV-infected (HIV [+]) women**

Parameters	hrHPV positive	hrHPV negative	P
	N = 390	N = 838	
**Age, years, mean ± SD**	38.9 ± 6.7	40.7 ± 6.5	< 0.001
**Age category, years, n (%)**			< 0.001
**Age 30 – 34**	130 (46.8)	148 (53.2)	
**Age 35 – 39**	100 (27.9)	259 (72.1)	
**Age 40 – 44**	83 (28.1)	212 (71.9)	
**Age 45 – 49**	44 (25.7)	127 (74.3)	
**Age 50 – 61**	33 (26.4)	92 (73.6)	
**CD4 cell count, n (%)**			0.03
**CD4 cell count, ≥500**	156 (30.4)	357 (69.6)	
**CD4 cell count, 350 – 499**	103 (29.8)	243 (70.2)	
**CD4 cell count, 200 – 349**	91 (33.6)	180 (66.4)	
**CD4 cell count, <200**	40 (45.5)	48 (54.5)	
**Age at sexual debut, years, n (%)**			0.27
**Age <14**	9 (30.0)	21 (70.0)	
**Age 14 – 25**	78 (30.2)	180 (69.8)	
**Age at first pregnancy,years ± SD**	19.6 ± 3.2	20.3 ± 3.7	< 0.01
**Lifetime sexual partners**			< 0.001
**1- 2 sexual partners**	167 (27.1)	449 (72.9)	
**≥ 3 sexual partners**	223 (36.4)	389 (63.6)	
**Number of children delivered, mean ± SD**	3.0 ± 1.6	3.1 ± 1.7	0.21
**Number of children delivered, n (%)**			0.15
**0 – 2**	150 (33.5)	298 (66.5)	
**3 – 4**	158 (34.0)	306 (66.0)	
**5 – 12**	59 (27.0)	160 (73.0)	
**Rape, n (%)**			
**Yes**	135 (33.0)	274 (67.0)	0.50
**No**	255 (31.1)	564 (68.9)	
**Menopause, n (%)**			0.07
**Yes**	27 (24.3)	84 (75.7)	
**No**	363 (32.5)	754 (67.5)	
**Polygamous relationship, n (%)**			0.22
**Yes**	13 (38.2)	21 (61.8)	
**No**	54 (27.8)	140 (72.2)	
**Ever used hormonal contraceptives, n (%)**			< 0.001
**Yes**	126 (41.3)	179 (58.7)	
**No**	264 (28.6)	658 (71.4)	
**Ever used tobacco, n (%)**			0.78
**Yes**	19 (33.3)	38 (66.7)	
**No**	369 (31.6)	800 (68.4)	
**Antiretroviral therapy, n (%)**			0.63
**Yes**	307 (32.4)	641 (67.6)	
**No**	82 (30.8)	184 (69.2)	
**Malaria within 6 months, n (%)**			0.34
**Yes**	26 (27.4)	69 (72.6)	
**No**	364 (32.1)	769 (67.9)	
**BMI, mean ± SD, kg/m** ^**^2**^	22.7 ± 3.6	22.6 ± 3.8	0.59

Chi-square test for categorical variables and ANOVA for continuous.

**Table 3:** **Baseline characteristics by high-risk human papillomavirus (HrHPV) status, restricted to HIV-uninfected (HIV [-]) women**

Parameters	HrHPV positive	HrHPV negative	P value
	N = 107	N = 1,204	
**Age, years, mean ± SD**	39.5 ± 7.1	42.3 ± 8.3	< 0.001
**Age category, years, n (%)**			0.01
**Age 30 – 34**	35 (11.7)	264 (88.3)	
**Age 35 – 39**	22 (7.9)	257 (92.1)	
**Age 40 – 44**	24 (10.1)	213 (89.9)	
**Age 45 – 49**	13 (6.1)	198 (93.9)	
**Age 50 – 61**	13 (4.6)	272 (95.4)	
**Age at sexual debut, years, n (%)**			0.77
**Age <14**	1 (6.2)	15 (93.8)	
**Age 14 – 25**	28 (8.3)	310 (91.7)	
**Age at first pregnancy,years ± SD**	20.5 ± 3.8	21.2 ± 3.7	0.07
**Lifetime sexual partners**			< 0.001
**1- 2 sexual partners**	99 (8.7)	1033 (91.3)	0.05
**≥ 3 sexual partners**	8 (4.5)	171 (95.5)	
**Number of children born, mean ± SD**	3.7 ± 1.6	3.9 ± 1.8	0.24
**Number of Children born, n (%)**			0.40
**0 – 2**	23 (7.7)	277 (92.3)	
**3 – 4**	46 (9.1)	458 (90.9)	
**5 – 12**	32 (6.8)	437 (93.2)	
**Rape, n (%)**			0.74
**Yes**	14 (7.5)	173 (92.5)	
**No**	92 (8.2)	1031 (91.8)	
**Menopause, n (%)**			0.03
**Yes**	10 (4.6)	209 (95.4)	
**No**	97 (8.9)	995(91.1)	
**Polygamous relationship, n (%)**			0.54
**Yes**	6 (10.0)	54 (90.0)	
**No**	46 (7.7)	547 (92.3)	
**Ever used hormonal contraceptives, n (%)**			0.81
**Yes**	33 (8.4)	360 (91.6)	
**No**	73 (8.0)	839 (92.0)	
**Ever used tobacco, n (%)**			0.42
**Yes**	15 (6.8)	206 (93.2)	
**No**	92 (8.4)	1004 (91.6)	
**Malaria within 6 months, n (%)**			0.77
**Yes**	14 (7.6)	171 (92.4)	
**No**	92 (8.2)	1032 (91.8)	
**BMI, mean ± SD, kg/m** ^**^2**^	21.9 ± 3.5	21.9 ± 3.7	0.98

Chi-square test for categorical variables and ANOVA for continuous.

**Figure 2:**
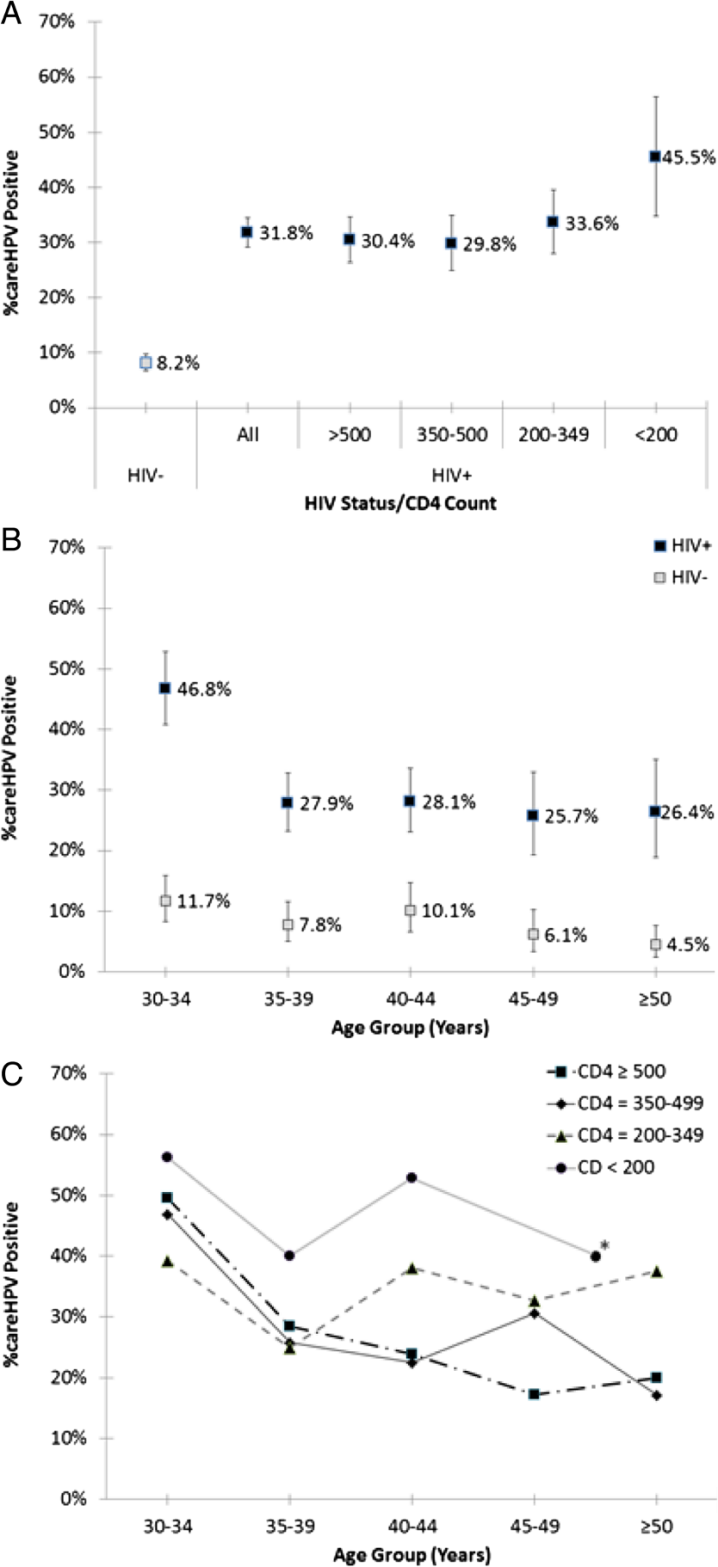
**Percent careHPV positive (high-risk HPV prevalence) by HIV status and CD4 count category (A), by age group for HIV status (B), and by age group and CD4 category among HIV+ women (C).** In Figure 1C, for CD4 < 200 (only), age groups 45-49 and 50+ years were combined due to small numbers (indicated by *).

The prevalence of hrHPV by HIV status and CD4 counts, by HIV status and age, and by HIV status, CD4 counts, and age are shown in Figure 2 (Panels A, B, and C, respectively). Overall, hrHPV prevalence was higher in HIV [+] women (31.8%, 95% CI = 29.2-34.4%) than HIV [-] women (8.2%, 95% CI = 6.7-9.8%) (p < 0.001). HIV [+] women who were more immune suppressed tended to have a greater prevalence of hrHPV (30.4%, 29.8%, 33.6% and 45.5% hrHPV prevalence for CD4+ cell count categories of ≥500, 350 – 499, 200-349 and <200 cells/μL, respectively) (p_trend_ <0.001) (Figure 2A). HrHPV prevalence declined with increasingly older age groups in HIV [+] women, from 46.8% in 30-34 years to 26.4% in 50-60 years (p_trend_ <0.001), and HIV [-] women, from 11.7% in 30-34 years to 4.5% in 50-60 years (p_trend_ <0.001) (Figure 2B). Among HIV [+], hrHPV prevalence declined with age for every category of CD4 cell count (Figure 2C).

Table 4 presents the results of the multivariate model for risk factors for hrHPV in HIV [+] women. A CD4 count of <200 cells/μL (vs. ≥500 cells/μL) was associated with testing hrHPV positive (adjusted Odds Ratio (aOR) = 2.21; 95% CI = 1.37 – 3.55) but CD4 counts of 200-349 or 350-499 cells/μL (Vs. ≥500 cells/μL) were not. Among HIV [+] women, age groups older than 30-34 years were independently approximately 2-fold less likely to test hrHPV positive compared to 30-34 year olds, but ART use was not independently associated with testing hrHPV positive. HIV [+] women with history of 3 or more lifetime number of sexual partners (vs. 1–2 lifetime number of sexual partners) were more likely to test hrHPV positive (aOR = 1.6; 95% CI = 1.2-2.0) while HIV [+] women with a history of using hormonal contraceptives were more likely to test hrHPV positive than those who did not (aOR = 1.5; 95% CI = 1.1–2.0).

**Table 4:** **Factors associated with high-risk human papillomavirus among HIV [+] women as determined using univariate and multivariate logistic regression**

Parameters	Unadjusted model		Adjusted model	
	Odds ratio	95% CI	Odds ratio	95% CI
**Age category, years, n (%)**				
**Age 30 – 34 (Ref.)**	1.0		1.0	
**Age 35 – 39**	0.44	0.32 – 0.61	0.44	0.31 – 0.61
**Age 40 – 44**	0.45	0.32 – 0.63	0.47	0.33 – 0.68
**Age 45 – 49**	0.39	0.26 – 0.60	0.42	0.27 – 0.65
**Age 50 – 61**	0.41	0.26 – 0.65	0.49	0.30 – 0.79
**CD 4 cell count, cells/ μL**				
**CD4 cell count, ≥500 (Ref.)**	1.0		1.0	
**CD4 cell count, 350 – 499**	1.0	0.74 – 1.3	1.0	0.76 – 1.4
**CD4 cell count, 200 – 349**	1.2	0.87 – 1.6	1.3	0.95 – 1.8
**CD4 cell count, <200**	2.0	1.2 – 3.1	2.2	1.4 – 3.6
**Lifetime sexual partners: ≥3 vs. 1-2**	1.5	1.2 – 2.0	1.6	1.2 – 2.0
**Ever used hormonal Contraceptives: Yes vs. No**	1.8	1.3 – 2.3	1.5	1.11– 2.0
**Ever used tobacco use, Yes vs. No**	1.1	0.62 – 1.9		
**Ever been raped, Yes vs. No**	1.1	0.85 – 1.4		
**Antiretroviral Treatment: Yes vs. No**	1.1	0.80 – 1.4		
**Number of children delivered**	0.95	0.88 – 1.0		
**Malaria within 6 months, Yes vs. No**	0.80	0.50 – 1.3		
**Menopause, Yes vs. No**	0.67	0.43 – 1.1		

Stepwise Logistic regression with entry and stay p = 0.1.

Table 5 presents the results of the multivariate model for risk factors for hrHPV in HIV [-] women. Only older age was independently (negatively) associated with testing hrHPV positive, with HIV [-] women aged 50-60 being the least likely to test hrHPV positive (aOR = 0.36; 95% CI = 0.19 – 0.69). Surprisingly, having 3 or more lifetime number of sexual partners (vs. 1-2 lifetime number of sexual partners) was negatively associated with testing hrHPV positive in HIV [-] women, although this finding was not statistically significant. However, few HIV [-] women reported having more than 3 or more lifetime number of sexual partners.

**Table 5:** **Factors associated with high-risk human papillomavirus among HIV [-] women as determined using univariate and multivariate logistic regression**

Parameters	Unadjusted model		Adjusted model	
	Odds ratio	95% CI	Odds ratio	95% CI
**Age category, years, n (%)**				
**Age 30 – 34 (Ref.)**	1.0		1.0	
**Age 35 – 39**	0.64	0.37 – 1.1	0.64	0.37 – 1.1
**Age 40 – 44**	0.85	0.49 – 1.5	0.85	0.49 – 1.5
**Age 45 – 49**	0.50	0.26 – 0.96	0.49	0.25 – 0.96
**Age 50 – 61**	0.36	0.19 – 0.70	0.36	0.19 – 0.69
**Lifetime sexual partners: ≥3 vs. 1-2**	0.49	0.23 – 1.0		
**Ever used hormonal Contraceptives: Yes vs. No**	1.1	0.69 – 1.6		
**Ever used tobacco use, Yes vs. No**	0.79	0.45 – 1.4		
**Ever been raped, Yes vs. No**	0.91	0.51 – 1.6		
**Number of children borne**	0.93	0.83 – 1.1		
**Malaria within 6 months, Yes vs. No**	0.92	0.51 – 1.7		
**Menopause, Yes vs. No**	0.49	0.25 – 0.96		

Stepwise Logistic regression with entry and stay p = 0.1.

## Discussion

We found a more three-fold higher prevalence of hrHPV infection in HIV [+] than HIV [-] Rwandan women. This finding is consistent with results from other studies from Africa and elsewhere [34, 36, 39, 42, 43]. In two previous Rwanda studies, a higher prevalence of hrHPV infection was found in HIV [+] than in HIV [-] Rwandan women for all age groups: (25 -34 years, 50% vs. 16%; 35 - 44 years, 42% vs. 8%; 45 -54, 33% vs. 5%) in the first study and (all ages, 50.8% vs. 31.8%) in the second study [7, 42].

However, hrHPV prevalence in HIV [+] women in this study was lower compared that reported from other studies of HIV [+] women [25, 27, 34, 42]. This may be due to the fact that this was a population-based study with community recruitment and a high proportion of women were on ART (78.1%), whereas other studies did not include women on ART [34, 44] or recruited mainly sexually high-risk populations [34, 42].

Among HIV [+] women included in this study, severe immunosuppression (CD4 cell count: <200 vs. ≥500 cells/μL) was independently associated with hrHPV infection in HIV [+] women; other investigators reported similar findings [34, 42, 45]. Because we only have prevalence data from our cross-sectional study, we cannot determine whether the effect of immunosuppression was to increase susceptibility of infection (incidence), the likelihood of persistence, and/or reactivation of quiescent infections [46]. To discriminate between these explanations and to understand the natural history of HPV and the associated risks for ≥ CIN3 in HIV [+] women living in Sub-Saharan Africa, large, well-powered cohort studies with longitudinal follow-up are needed.

Our study also found that among HIV [+] women hormonal contraception was independently associated with hrHPV infection. It is unclear if contraception was a marker for more risky sexual behavior and condom non-use or could be otherwise directly causal through hormones. But our finding with regard to the association hrHPV and hormonal contraception was inconsistent with several studies [47–50] including a study in 375 HIV-infected Canadian women, in which there was no association with hormonal contraceptive use and hrHPV prevalence. Given the magnitude of association of hormonal contraceptives in the present study and the high use rate of hormonal contraception in Africa, more studies of African HIV [+] women are needed to clarify this relationship.

Although we did not compare HIV [+] and HIV [-] women directly in a multivariate model due to differences in the risk factor profiles for the two populations, HIV infection appears to be an independent risk factor for hrHPV, as even HIV [+] women with CD4 counts of ≥500 cells/mL had a more than 3-fold greater hrHPV prevalence than HIV [-] women. A similarly high magnitude of association of HIV infection with hrHPV was previously found in a Ugandan population: aOR = 4.82, 95% CI = 3.10-7.53 [51]. Two other studies previously showed that HIV-infected women are more likely than those without HIV infection to have persistent HPV [52, 53]. But with respect to HPV natural history, there is an incomplete re-constitution of immune response to HPV with ART. ART itself was not an independent predictor of hrHPV prevalence but its effects were likely mediated through CD4 counts.

Although prophylactic HPV vaccines may prove to be the ultimate cervical cancer prevention strategy, there are already 2-3 generations of at-risk, hrHPV-positive women who will not benefit from HPV vaccination, and universal HPV vaccination is decades away. Moreover, in the ART era, HIV [+] women will live longer, have a greater hrHPV burden, and in the absence of comprehensive screening and treatment, will likely remain at an elevated risk of invasive cervical cancer. Thus, there is still a need to better understand what comprises an effective immune response against HPV infection. Such research may provide important clues to the development of effective biological therapeutics against HPV and HPV-related disease that might be used to prevent HPV-related cancers in those already infected with hrHPV. Our study had limitations mainly due to the cross sectional design with potential problems with direction of causality, which means that HPV could possibly increase the likelihood of HIV infection or vice versa. Recent evidence suggests that HPV infection can increase the risk of HIV acquisition [54].

In conclusion, we found that HIV [+] women had a higher hrHPV prevalence than HIV [-] women, and that the hrHPV prevalence in HIV [+] women was inversely related to immune suppression as measured by CD4 cell counts. Our data have important implications for hrHPV-based screening in HIV [+] women living in Rwanda and elsewhere. In the general population, hrHPV-based screen and treat, in which all hrHPV-positive women are treated immediately, may be a reasonable strategy, given the relatively few women who will be treated and the lack of colposcopist and pathologists [55] to provide tissue-based diagnosis to guide treatment. However, in the HIV [+]women, especially those who are highly immune suppressed and are likely to be at the greatest risk of cervical cancer [56] and [57], hrHPV-based screening may be to non-specific to use alone, especially in a screen-and-treat strategy. In the Rwandan context, half of these highly immune suppressed women would test hrHPV positive and be treated. In other populations, an even higher proportion would be treated [25, 27, 34, 42]. Thus, it may be desirable to use a secondary, triage test to determine which hrHPV-positive women need immediate treatment (triage positive) and which might have treatment deferred (triage negative) until there is evidence of hrHPV persistence, which is a strong risk factor for cervical precancer and cancer [12, 13].

Although VIA has been proposed, as mentioned, VIA as a triage of hrHPV positives may significantly reduce the overall sensitivity of screening [29, 30]. Alternatively, very specific biomarkers, such as HPV E6 oncoprotein [32, 58] might be considered but has not been evaluated in this population.

Further investigations are need to determine the optimal cervical cancer screening strategies, those that balance the benefits and harms of screening, in known HIV [+] women living in Africa.
